# Association between social capital and oral health among adults aged 50 years and over in China: a cross-sectional study

**DOI:** 10.1186/s12903-022-02102-8

**Published:** 2022-03-12

**Authors:** Suyang Li, Yanfei Guo, Junmei Miao Jonasson

**Affiliations:** 1 School of Public Health and Community Medicine, Institute of Medicine, Sahlgrenska Academy, University of Gothenburg, Gothenburg, Sweden; 2 Shanghai Municipal Center for Disease Control and Prevention, Shanghai, China; 3 Department of Research and Development, Region Halland, Halmstad, Sweden

**Keywords:** Ageing, Social capital, Oral health, Edentulism

## Abstract

**Background:**

Social capital has a potential effect in protecting oral health among population. However, no study has explored the association between social capital and oral health in the Chinese context. Due to the unique culture, political, social context in China, it is important to understand their association in the Chinese context. The study aims to investigate the association between cognitive and structural dimensions of social capital with edentulism among adults aged 50 years and over in China.

**Method:**

The study used data from the WHO SAGE (Study on Global AGEing and Adult Health) wave 1 China component. Structural social capital was operationalized as social participation. Cognitive social capital was operationalized as perceived community trust and perceived community safety. Community-level social capital was measured by aggregating individual-level social capital into community level. Oral health was measured using a final marker of oral health status, self-reported edentulism. A 2-level multilevel logistic regression was used to evaluate the association between different dimensions of social capital and oral health.

**Results:**

In total, 12,856 individuals were included in the study, the overall prevalence of edentulism was 9.1% (95% CI 8.3–10.0). Multilevel logistic analysis revealed that individual-level social capital and community-level social capital are independently associated with edentulism. Individuals with low structural social capital and living in areas with low structural social capital have, respectively, 1.54 (95% CI 1.18–2.01) and 2.14 (95% CI 1.47–3.12) times higher odds for edentulism, after adjustment for potential confounders (age, sex, marital status, residence locality, wealth, education level, chronic conditions) and a potential mediator(smoking).

**Conclusions:**

Living in a community with lower structural social capital and individual with low structural social capital is associated with higher risk for edentulism among adults aged 50 years and over in China.

## Background

According to Global Burden of Disease in 2017, there are nearly 3.5 billion population affected by oral diseases around the world [1]. Oral health is an important public health issue globally, including in China. Among the Chinese population oral diseases is very common, particularly among older adults. According to the 4th national oral health epidemiological survey in China, the prevalence of dental caries and periodontal disease among the age group 55–74 were 96.8% and 92.9%, respectively [2]. These oral diseases are the main reason for tooth loss among Chinese population [3]. Factors that increase the risk for caries and periodontal diseases highly relate to health behaviors, such as, smoking, drinking, oral hygiene, dental attendance, eating food high in sugar [4–6]. Edentulism, which refers to the loss of all natural teeth, is a final status for tooth loss and is a good indicator of lifelong exposure to oral health risk factors. According to the 4th survey, the prevalence of edentulism aged 45 and above increased from 12.1% in 2013 to 14.6% in 2015 [3].

Traditionally, studies exploring possible determinants of oral health have paid most attention to biological and behavioral factors [7, 8] However, health behavior is also determined by a range of sociopolitical factors, for example, social capital [9]. Evidence has shown that social capital, which is defined by Putnam as “features of organizations, such as networks, trust and social norms of reciprocity” [10], is associated with oral health in different contexts [11–13]. For example, in Brazilian, a higher level of neighborhood social capital was reported to have an association with lower risk of dental caries [14] and lower risk of dental injury [15] among adolescence. In the US, a study found that more numbers of close friends is associated with fewer decayed teeth among individuals age 60 and above [16]. In Japan, participation in hobby clubs, sports groups was associated with reduced risk of tooth loss among older people[17]. The association between social capital and oral health was also found in other countries, including England [18], Indonesia [19, 20], South African [21], Korea [22], India [23]. Although studies have explored the association between social capital and different health outcomes in the Chinese context, including, loneliness [24, 25], depression [26–28], life satisfaction [29], self-rated health [30–32], mental health [33, 34], health-related quality of life [35, 36], cognitive functions [37], however, no study has explored its association with oral health. Due to different epidemiological characteristics of oral health conditions and differences in political, socioeconomic, cultural context, and health care system in China, social capital may have a different effect on oral health. Therefore, it is important to explore the association in the Chinese context.

In addition, to explore the mechanism between social capital and oral health, it is important to understand the association of different dimensions of social capital with oral health. Islam developed a very useful division for social capital and broke social capital into two dimensions: cognitive social capital and structural social capital [11]. Cognitive dimension of social capital refers to individuals’ subjective perception of the community or society the individuals belong to, while structural social capital refers to the actual behavior to build social networks [38]. Giordano and colleagues suggested that researchers should both explore the cognitive and structural dimensions of social capital [39], due to it may impacts health through different pathways [40]. Structural social capital is shaped by institutions, policies, and culture, which potentially have strong impact on objective health of individuals, while cognitive social capital is suggested to affect health through psychosocial mechanisms and potentially have impact on subjective health of individuals [40].

Therefore, the present study aims to explore the association of cognitive and structural social capital at the individual- and community-level and edentulism among adults aged 50 and over in China.

## Methods

### Study sample and design

SAGE(Study on Global AGEing and Adult Health) was a stratified, multistage sampled longitudinal cohort study conducted by WHO. It investigated individuals aged 18 years and over with a focus on individuals aged 50 and over in six developing countries. Data from SAGE wave 1 China component 2007–2010 was used in this study. Sample was stratified by 8 nationally representative provinces. A total of 64 townships and community blocks was selected from the 8 provinces using probability proportional to size method, and then, 127 villages and neighborhoods was selected from the townships and community blocks using probability proportional to size method. From each villages and neighborhoods, two residential blocks were selected using cluster random sampling. Altogether 15,050 individuals were randomly sampled from the residential blocks using a face-to-face questionnaire for data collection. The response rate for the questionnaire was 98% according to the working sheet of SAGE. Villages in rural areas and neighborhoods in urban areas, which are administrative boundaries with a similar population density, were defined as communities in this study. The study is a cross-sectional study with a focus on individual respondents aged 50+ years. Thus, individuals who aged below 50 years and had missing data for the outcome variable were excluded. The final sample size for the study is 12,856 [41].

### Variables

#### Outcome variable

Self-reported edentulism was the dependent variable. Respondents were asked the question: “Have you lost all of your natural teeth?” Participants who answered “yes” were coded as edentulism (lose all natural teeth).

#### Main exposure variables

Individual social capital was measured using a social-cohesion based approach which is based on Putnam’s definition of social capital [10]. The approach is most widely used in public health. It inquires the potential availability of resources in the group (e.g. social participation, trust) instead of inquiring individual’s social network connections (e.g. number of close ties) [30]. Cognitive social capital was measured by two dimensions: perceived community trust and perceived community safety through 4 questions. Two questions about trust in different groups of people were asked. Participants were asked, “generally speaking, would you say that you can trust: (1) your neighbors; (2) co-workers?” The answers were a 5-item scale from 1 = to a very great extent to 5 = to a very small extent and were reverse coded as 1 = to a very small extend to 5 = to a very great extend. Perceived community safety was measured by asking 2 questions, “How safe from crime and violence do you feel when you are alone at home?” and “How safe do you feel when walking down your street alone after dark?” The answers were from 1 = completely safe to 5 = not safe at all and the answers were reverse coded. The final cognitive social capital score was the sum of the scores of perceived community trust and perceived community safety with a range from 4 to 20 (Cronbach alpha = 0.6638). Cognitive social capital score was divided into tertile, higher score indicates higher social capital.

Structural social capital, which refers to the actual behavior to build social network, was measured by assessing the frequency of social participation, including formal social participation (e.g. participate in public activities like school, public activities) and informal social participation (e.g. participate leisure activities with friends, families). It was measured by 9 questions that indicate the frequency of community activities involved in the past 12 months, including, how often in the last 12 months (1) have you attended public meetings; (2) attended religious services; (3) attended social meetings, programs, activities, or events or to visit friends or relatives; (4) attending any group, club, society, union, or organization meeting; (5) meeting personally with a community leader; (6) interacting with neighbors; (7) having friends over to home; (8) been in the home of someone living in a different neighborhood; (9) socializing with co-workers. The score was summed up to represent the total score of individual structural social capital, with a range from 9 to 45 (Cronbach alpha = 0.6246). Then, the score was divided into tertiles, namely, high, middle, low.

Community-level cognitive social capital was measured by aggregating individual cognitive social capital into group level. The mean value of cognitive social capital in each community were calculated and centered. Based on the centered mean value of cognitive social capital of each community, these communities were grouped into tertile, namely, high-, intermediate- and low community level cognitive social capital. Community-level structural social capital was measured in the same way. Totally 127 communities were used in calculating the community-level social capital. This method was prevalent being used in measuring group-level social capital by previous studies [42–44].

#### Covariates

Data on demographic variables were collected, including sex, age, highest education, marital status, locality( rural or urban), and household income. Age was grouped into 4 groups: 50–59 years, 60–69 years, 70–79 years, and 80- years. According to the highest education obtained, participants also were categorized into 6 groups: no formal education, primary school uncompleted, primary school completed, secondary school completed, high school completed, and college or above. Marital status was dichotomized into two groups including married (currently married or cohabited) and unmarried (never married, divorced, separated, and widowed). The locality was grouped into two categories including rural and urban areas based on the participants’ address at which the data was collected. Household income was categorized into 5 quintiles depended on dwelling characteristics and asset possessions calculated by principle components analysis provided by the data [45].

Seven common chronic diseases including angina, stroke, diabetes, chronic lung diseases, asthma, hypertension, and depression were assessed through self-report. Participants were asked, “ have you ever been diagnosed with the above chronic conditions?” The answers were yes or no. A total number of chronic diseases was calculated.

Smoking status was measured by two questions: “Do you ever smoked tobacco or used smokeless tobacco? (yes or no)” and “Do you currently use any tobacco products such as cigarettes, cigars, pipes, chewing tobacco or snuff? (yes daily; yes, not daily; no, not at all)”. Based on the two questions participants were categorized into four groups: non-smoker, former smoker, current smoker (not daily), and current smoker (daily).

### Method of analysis

**Fig. 1:**
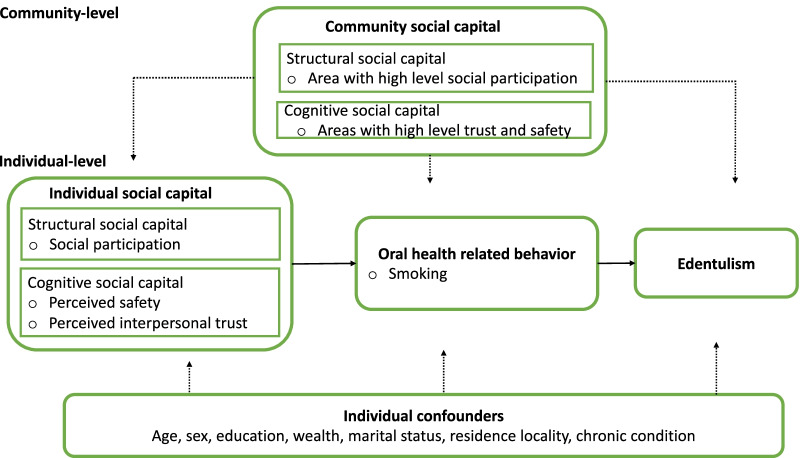
Conceptual model of individual and community social capital on edentulism

Statistic analyses were operated in Stata SE version 16.0 (Stata Corp, College Station, TX). The weights calculated by selection probability and post-stratification were standardized by dividing each weight by the mean weights of the sample. The commands “svy” of Stata were used to incorporate weight into the analysis. Descriptive statistic was used to describe the characteristics of the study population, including age, sex, residence locality, educational level, marital status, household income, behavioral factors, and chronic health conditions, adjusted by standardized weights. A 2-level random intercept, fixed slope multilevel logistic model was used to evaluate the association between two dimensions of social capital and edentulism. Crude odds ratios were calculated by univariate multilevel logistic analysis. A conceptual model based on Rouxel [46] and Watt [47] was developed to test association between social capital and edentulism in Fig. 1. A series of multilevel logistic models were established. Initially, we included community level social capital (model 1), then we added individual level social capital (model 2), followed by adjusting for potential confounders (model 3), and, finally, we included a potential mediator, oral health-related behaviour (smoking), in model 4. The Statistic analysis were estimated with a 95% confidence interval. A significant level was set at *p* value $<0.05$.

### Ethical consideration

SAGE was approved by the Ethical Review Board of WHO (RPC146), and the ethical review committee of China (approval notice 200601). Informed consent was obtained from all participants.

## Results

The socioeconomic, demographic, behavioral characteristics, and chronic health condition of the study sample were illustrated in Table 1. A total of 12,856 individuals aged 50 and above were included in the analysis. The overall average age of 12,856 respondents in 127 communities was 63.0 (standard deviation; 9.3) years old. The overall prevalence of participants being edentulate was 9.1% (95% CI 8.3–10.0). The majority of the participants of the sample population were between 50 to 69 years old (77.3%), currently married (85.4%), completed primary school and above (58.7%), never smoker (64.1%), and never drinker (66%), sufficient fruit or vegetable intaking (90.1%), having no chronic diseases (61%). Nearly half (47.6%) of the participants lived in urban areas. The proportion of highest and lowest wealth quintile were, respectively, 21.9% and 16.1%.

**Table 1:** Sociodemographic characteristics of the study population (n = 12,856)

Variables	N (%) weighted	Variables	N (%) weighted
*Sex*		*Marital status*	
male	6393 (49.8)	Married	10951 (85.4)
female	6445 (50.2)	Unmarried	1878 (14.6)
*Age*		*Numbers of chronic diseases*	
50-59	5803 (45.2)	No chronic diseases	7844 (61.1)
60-69	4104 (32.0)	1 chronic disease	3415 (26.6)
70-79	2376 (18.5)	2 chronic diseases	1220 (9.5)
80-	554 (4.3)	3–6 chronic diseases	347 (2.7)
*Residence*		*Smoking status*	
Urban	6111 (47.6)	Never smoker	8195 (64.1)
Rural	6727 (52.4)	Not current smoke	844 (6.6)
*Education*		Current smoker, not daily	320 (2.5)
No formal education	2855 (22.2)	Current daily smoker	3426 (26.8)
Less than primary	2455 (19.1)		
Primary completed	2727 (21.2)		
Secondary completed	2577 (20.1)		
High school completed	1640 (12.8)		
University and above	585 (4.6)		
*House household income quintile*			
Q1 (lowest)	2056 (16.1)		
Q2	2309 (18.0)		
Q3	2617 (20.5)		
Q4	3008 (23.5)		
Q5 (highest)	2805 (21.9)		

Table 2 shows the distribution of edentulism and the crude odds ratio of covariables for edentulism. Descriptive statistics show that the prevalence of edentulism is higher among females (10.1%) than males (8.0%). The age group 80+ has the highest prevalence of edentulism (37.1%). Those living in rural areas (10.4%) have higher prevalence of edentulism than those living in urban areas (7.6%). Compared with those married (7.6%), those unmarried (17.6%) have higher prevalence of edentulism. Those have lower educational level and in lower household income quintile have higher prevalence of edentulism than the counterpart. Crude odds ratio of covariables (Table 2) were calculated by univariate multilevel logistic regression analysis. The crude odds ratio of covariates identified variables that were associated with edentulism. Edentulism was significantly associated with being females, oldest age group, no formal education, unmarried, lowest household income, living in rural area, smoking, and having one chronic diseases.

**Table 2:** Distribution of covariables by edentate status and crude odds ratio of covariables for edentulism (n = 12,856)

Co-variables	Non-edentate (% weighted)	Edentate (% weighted)	Crude odds ratio (95% CI)
*Sex*			
male	5878 (92.0)	514 (8.0)	Reference
female	5795 (89.9)	653 (10.1)	1.32 (1.16–1.50)***
*Age*			
50-59	5639 (97.2)	164 (2.8)	Reference
60-69	3757 (91.5)	347 (8.5)	3.36 (2.70–4.18)***
70-79	1925 (81.0)	545 (19.0)	9.94 (7.64–12.91)***
80-	349 (62.9)	206 (37.1)	24.20 (17.11–34.22)***
*Residence*			
Urban	5647 (92.4)	464 (7.6)	Reference
Rural	6027 (89.6)	700 (10.4)	1.49 (1.12–1.98)**
*Education*			
No formal education	2350 (82.3)	505 (17.7)	6.83 (4.12–11.32)***
Less than primary	2200 (89.6)	255 (10.4)	3.51 (2.12–5.81)***
Primary completed	2522 (92.5)	205 (7.5)	2.30 (1.36–3.88)**
Secondary completed	2448 (95.0)	129 (5.0)	1.49 (0.91–2.45)
High school completed	1587 (96.8)	52 (3.2)	0.95 (0.56–1.59)
University and above	565 (96.7)	19 (3.3)	Reference
*Household income quintile*			
Q1 (lowest)	1740 (84.6)	316 (15.4)	3.69 (2.69–5.05)***
Q2	2057 (89.1)	252 (10.9)	2.16 (1.52–3.07)***
Q3	2349 (89.8)	267 (10.2)	1.91 (1.41–2.59) ***
Q4	2812 (93.5)	196 (6.5)	1.31 (0.94–1.82)
Q5 (highest)	2669 (95.1)	137 (4.9)	Reference
*Marital status*			
married	10114 (92.4)	837 (7.6)	Reference
unmarried	1548 (82.4)	322 (17.6)	2.61 (2.21–3.07)***
*Smoking status*			
Never smoker	7434 (90.8)	755 (9.2)	Reference
former smoker	729 (86.4)	115 (13.6)	1.59 (1.23–2.06)***
Current smoker, not daily	303 (94.4)	18 (5.6)	0.59 (0.36–0.96)*
Current daily smoker	3158 (92.1)	272 (7.9)	0.79 (0.66–0.95)*
*Numbers of chronic diseases*			
No chronic diseases	7246 (92.4)	598 (7.6)	Reference
1 chronic disease	3076 (89.9)	344 (10.1)	1.39 (1.16–1.67)***
2 chronic diseases	1056 (86.2)	170 (13.8)	2.16 (1.73–2.71)***
3-6 chronic diseases	291 (83.8)	56 (16.2)	2.68 (1.86–3.85)***

***$$p<0.001$$, **$$p<0.01$$,*$$p<0.05$$

Table 3 shows crude odds ratio of social capital variables for edentulism calculated by uni-variate multilevel logistic regression. At individual level lowest cognitive and structural social capital was,respectively, associated with 1.34 (95% CI 1.08–1.67) and 2.44 (95% CI 1.92–3.11) times higher odds of edentulism compared with highest social capital group. At community level, lowest cognitive and structural social capital was, respectively, associated with 1.18 (95% CI 0.83–1.66) and 1.81 (95% CI 1.28–2.58) times higher odds of edentulism compared with highest social capital group. Table 4 shows adjusted and unadjusted odds ratio of social capital variables for edentulism. Model 0 was a null model including no explanatory variable, which is baseline for community differences. In model 1, community-level social capital was included, individuals living in low structural social capital areas have higher odds for edentulism. In model 2, individual and community level social capital was included. Individual-level low structural social capital was significantly associated with edentulism with odds of 2.39 (95% CI 1.86–3.08). No significant association was found between community social capital variables, individual cognitive social capital and edentulism. After adjusting for potential confounders (model 3), including socioeconomic factor, demographic factors and chronic condition, only structural dimension of individual and community social capital were significantly associated with edentulism, the odds were respectively, 1.54 (95% CI 1.18–2.02) and 2.11 (95% CI 1.45–3.07). In the final model (model 4), further include a potential mediator (smoking), the structural dimension of both level social capital is still significantly associated with edentulism. The intraclass correlation for model 0 was 13.53%, which indicated that 13.53% of individual health variance was attributable to the community differences. After adjusting for all explanatory variables in final model, intraclass correlation reduced to 10.93%, indicating the model fits better.

**Table 3:** Crude odds ratios (OR) of social capital variables for edentulism

Main exposure variables	Edentulism
	Crude OR (95% CI)
*Individual cognitive social capital*	
Low	1.34 (1.08–1.67)**
Mediate	1.02 (0.82–1.27)
High	Reference
*Individual structural social capital*	
Low	2.44 (1.92–3.11)***
Mediate	1.52 (1.25–1.84)***
High	Reference
*Community cognitive social capital*	
Low	1.18 (0.83–1.66)
Mediate	1.21 (0.84–1.75)
High	Reference
*Community structural social capital*	
Low	1.81 (1.28–2.58)**
Mediate	1.07 (0.79–1.46)
High	Reference

***$$p<0.001$$, **$$p<0.01$$, *$$p<0.05$$

**Table 4:** Multilevel logistic regression model for edentulism unadjusted and adjusted by covariates

Fixed effects	Model 0^a^	Model 1^b^	Model 2^c^	Model 3^d^	Model 4^e^
		OR (95%CI)	OR (95%CI)	OR (95%CI)	OR (95%CI)
		Unadjusted	Unadjusted	Adjusted	Adjusted
* Individual cognitive SC*^f^					
Low			1.17 (0.96–1.44)	1.13 (0.91–1.41)	1.14 (0.91–1.43)
Mediate			0.93 (0.76–1.14)	0.91 (0.74–1.11)	0.92 (0.75–1.12)
High			Reference	Reference	Reference
* Individual structural SC*^f^					
Low			2.39 (1.86–3.08)$$^{***}$$	1.54( 1.18–2.02)$$^{**}$$	1.54 (1.18–2.01)$$^{**}$$
Mediate			1.48 (1.20–1.82)$$^{***}$$	1.25 (1.00–1.57)$$^{*}$$	1.24 (0.99–1.55)
High			Reference	Reference	Reference
* Community cognitive SC*^f^					
Low		1.08 (0.76–1.53)	1.00 (0.70–1.44)	1.05 (0.74–1.50)	1.03 (0.72–1.48)
Mediate		1.12 (0.78–1.61)	1.08 (0.76–1.55)	1.02 (0.71–1.47)	1.01 (0.70–1.45)
High		Reference	Reference	Reference	Reference
* Community structural SC*^f^					
Low		1.77 (1.24–2.53)**	1.37 (0.94–1.99)	2.11 (1.45–3.07)***	2.14 (1.47–3.12)***
Mediate		1.04 (0.76–1.43)	0.91 (0.66–1.27)	1.10 (0.79–1.54)	1.11 (0.79–1.55)
High		Reference	Reference	Reference	Reference
*Random effects*					
Community variance (SE)	0.515 (0.097)	0.449 (0.091)	0.469 (0.095)	0.397 (0.083)	0.403 (0.085)
Intraclass correlation (%)	13.53%	12.02%	12.49%	10.78%	10.93%

***$$p<0.001$$, **$$p<0.01$$, *$$p<0.05$$

^a^Model 0 was a null model including no explanatory variable

^b^Model 1 include community level social capital variables

^c^Model 2 include individual level and community level social capital variables

^d^Model 3 adjusted for age, sex, marital status, education attainment, residence locality, wealth quintile and chronic condition

^e^Model 4 adjusted for model 3 plus smoking status

^f^SC refers to social capital

## Discussion

The present study investigated the association of cognitive and structural social capital at both individual- and community-level and edentulism among adults aged 50 and over in China. After adjusting for potential confounders and a potential mediator, individual with low structural social capital and living in areas with low structural social capital was independently associated with higher odds for edentulism. While cognitive dimension social capital was not associated with edentulism in this study. The result indicates that the structural dimension of social capital has a larger influence on edentulism than the cognitive dimension among Chinese adults aged 50 years and over.

At the individual level, the result of the association between structural social capital and edentulism is consistent with the previous study by Rouxel et al, who conducted a cross-sectional study of 8552 individuals aged 50 years and older in the English population and found that structural social capital—as measured by volunteer status and membership in organizations—was associated with edentulism [40]. Also, a cross-sectional study conducted in Korea found that having less than one general social network (structural social capital) was associated with higher risk of poor chewing ability among the old population aged 70 and over in the rural area. Also, other studies that didn’t differentiate the cognitive and structural social capital found a consistent association between individual structural social capital and the oral health measured by number of remaining teeth [17, 43, 48, 49]. Despite these studies used different measurements for structural social capital, their findings are consistent. This study didn’t find an association between individual cognitive social capital and edentulism. However, cognitive social capital is often associated with subjective oral health indicators reported by other studies, such as self-reported oral health [18, 40, 50]. This inconsistency in the association between individual cognitive social capital and oral health might be that cognitive social capital impact oral health through psychosocial pathway and was suggested affect individual’s subjective health (e.g. self-reported oral health), rather than objective oral health measurement (e.g. edentulism).At community level, the present study found that it is the structural dimension of social capital, not the cognitive dimension, is associated with higher odds of edentulism. This finding is also consistent with a previous study. Koyama and colleagues conducted a longitudinal cohort study on community social capital and tooth loss and found that civic participation at baseline is associated with dental loss among Japanese older adults, but not community trust and attachment [17].

The mechanism between social capital and health is not fully understood. There are several hypothesized ways linking social capital and oral health that may explain our findings. Firstly, social capital may impact oral health through a psychosocial pathway, and it has been reported that individual with lower social capital is vulnerable to psychosocial stressors, thus, deteriorate immune system and ability of the body to defence oral bacteria [51]. Also, psychosocial stressors may trigger coping mechanisms related to health-compromising behaviors which increase the risk of dental caries and periodontal diseases, such as, smoking, eating sweet food, drinking alcohol [52]. In addition, social capital can impact oral health through increasing access to dental health care. For example, friends or family members could help to drive them to a hospital or make a dental appointment for them. Moreover, at community level, individuals can benefit from being members of a community. A closely connected community has high level of collective efficiency, which can promote collective action. For example, lobby local authorities for increasing access to health care. Another group-level mechanism is informal social control, which is the ability of a community to maintain social order. In a cohesive community, members are more likely to intervene when witnessing misbehavior, such as health-compromising behavior. Moreover, community social capital can impact health through social contagion. For instance, oral health-related information or behavior norms spread more quickly in a tight-knit community.

In the theoretical framework, oral health related behaviour, smoking, was included as a potential mediator. Previous studies found that social capital is associated with smoking [53]. In our study, smoking is significantly associated with edentulism in the final model. Also, smoking was found a strong association with oral health reported by other study [54]. Thus, these associations suggested the potential mediator effect of smoking on association between social capital and oral health. Besides, other oral health related behaviors, like eating sweet and access to dental health care also are potential mediators of interest. Future studies are needed to further explore their mediator effect to better understand the mechanism between social capital and oral health.

This study have explored the association of cognitive and structural dimensions of social capital and oral health in Chinese context. It is important to understand the influence of social capital in the Chinese context. Firstly, as a result of dental health care is expensive in China, and most of the cost is not covered by medical insurance, which increase the importance of the role of social capital. In addition, due to high state ownership in China, government officials in China have control on more resources than those in other countries, which means that having relationship with government officials may receive more potential benefit [55]. Lastly and most importantly, China had long time implemented “one child policy”, which increased the number of older population living alone and reduced the number of close ties (having one child) of older population [56]. Those unique context make China an important research object. Despite this study have explored association between cognitive and structural dimension social capital and edentulism. It is also important to understand the influence of other forms of social capital in the Chinese context. For example, some studies found that different dimensions of social capital play a different role in oral health. For example, in Japan, a cross-sectional study found that horizontal social capital, which refers to relationship ties that exist among equal individual or groups who have equal or similar access to power or resources, play a more important role in oral health among the old population than vertical social capital [43]. Moreover, a cross-sectional study of 967 students aged 18 and 19 years showed that it was vertical trust (trust between students and teachers), but not horizontal trust (trust between students), was associated with poor self-reported oral health [57]. Additionally, among the Brazilian population, a multilevel cross-sectional study found that bonding social capital was associated with dental pain, but not bridging social capital [44]. Understanding which form of social capital has a more important effect on oral health can help to develop a more effective intervention for oral health.

One strength of the present study is that we both assessed individual- and community-level social capital using multilevel perspective. Social capital is both characteristics of individual and community [38]. An individual can mobilize community resources through their individual network. Another strength of the study is that it used a relatively large sample size and the data were nationally representative of community-dwelling aged 50 and above in China. In addition, the data was face-to-face collected using a valid questionnaire. The questionnaire had been tested by a polit study in three developing countries (Ghana, India and the Republic of Tanzania) and performed well with good face validity [58]. However, there were some limitations of the study. First, it was a cross-sectional study. We don’t know temporal sequencing of social capital and edentulism. Thus, we can not make a causal inference. In addition, we can not rule out reverse causality between social capital and edentulism. For example, edentulism may lead to health selection. Individuals with bad oral health may reduce social participation due to embarrassment caused by teeth. A future prospective study is needed to further explore the relationship between social capital and oral health. Second, information on use of prosthesis was lacking in SAGE data, which might be a potential factor that affect social participation. Future study is needed to take into account the use of prosthesis of edentulism individuals. In addition, information on edentulism was collected by self-report which may introduce information bias and social desirability bias. However, edentulism is not a sensitive issue in aged 50 and older adults in Chinese population and collected face-to-face by well-trained interviewers. The bias was considered to be minimal in our study. Third, the questions used for measurement of social capital may not have captured the whole dimension of social capital, because of the concept of social capital is still controversary, which inevitably result in divergent measurements and controversial operationalisations. Despite there is no best measurement for social capital, we used measurement commonly used by previous studies (social participation, trust, safety) [59]. The internal consistency of the scales we used for measuring social capital was not perfect, but was adequate with a value of Cronbach alpha above 0.6 [60]. Fourth, the measurement of individual-level trust in the present study, which is commonly used by previous studies for measurement of social capital [59], cannot distinguish between the actual trustworthiness of others and an individual’s tendency to trust others. We were interested in whether interpersonal trust or a trustworthy environment have a protective effect on oral health, not an individual’s tendency to trust. Thus, an individual’s variance of the tendency to trust may have biased the result of the study. Finally, the construction of community variables from individual level is one limitation. Community level social capital were measured by aggregating data, which may risk multilinearity due to variables were from same data source. Mean centering was used in our study to reduce multilinearity caused by aggregating data.

## Conclusions

This study evaluates the association of both cognitive and structural dimensions of social capital and edentulism at both individual- and community-level. It provides evidence that individual-level social capital and community-level social capital were independently associated with edentulism among the old adults in China. Individuals with low structural social capital and living in low structural social capital areas have higher risk for edentulism. In addition, the structural dimension of social capital may play a more important role in edentulism among adults aged 50 yearrs and over in China. Due to the protective role in health, social capital can be considered as a potential tool to promote oral health among Chinese population.

## Data Availability

The datasets supporting the conclusions of this article are available upon request in the website of WHO (https://apps.who.int/healthinfo/systems/surveydata/index.php/catalog/13).

## Abbreviations

SAGE: Study on Global AGEing and Adult Health
WHO: World Health Organisation
CI: Confidence interval
OR: Odds ratio
